# Genetics of validated Parkinson’s disease subtypes in the Oxford Discovery and Tracking Parkinson’s cohorts

**DOI:** 10.1136/jnnp-2021-327376

**Published:** 2022-06-21

**Authors:** Michael Lawton, Manuela MX Tan, Yoav Ben-Shlomo, Fahd Baig, Thomas Barber, Johannes C Klein, Samuel G Evetts, Stephanie Millin, Naveed Malek, Katherine Grosset, Roger A Barker, Nigel Williams, David J Burn, Thomas Foltynie, Huw R Morris, Nicholas Wood, Donald G Grosset, Michele Tao-Ming Hu

**Affiliations:** 1 Population Health Sciences, University of Bristol Medical School, Bristol, UK; 2 Department of Clinical and Movement Neurosciences, Queen Square Institute of Neurology, University College London, London, UK; 3 UCL Movement Disorders Centre, University College London, London, UK; 4 Nuffield Department of Clinical Neurosciences, University of Oxford, Oxford, UK; 5 Molecular and Clinical Sciences Institute, St. George’s University of London, London, UK; 6 Oxford Parkinson’s Disease Centre, University of Oxford, Oxford, UK; 7 Department of Physiology, Anatomy and Genetics, University of Oxford, Oxford, UK; 8 Department of Neurology, Queen's Hospital, Romford, Essex, UK; 9 Department of Neurology, Institute of Neurological Sciences, Queen Elizabeth University Hospital and University of Glasgow, Glasgow, UK; 10 Cambridge Centre for Brain Repair, University of Cambridge, Cambridge, UK; 11 Psychological Medicine and Clinical Neurosciences, Cardiff University, Cardiff, UK; 12 Faculty of Medical Sciences, Newcastle University, Newcastle, UK

**Keywords:** PARKINSON'S DISEASE, GENETICS

## Abstract

**Objectives:**

To explore the genetics of four Parkinson’s disease (PD) subtypes that have been previously described in two large cohorts of patients with recently diagnosed PD. These subtypes came from a data-driven cluster analysis of phenotypic variables.

**Methods:**

We looked at the frequency of genetic mutations in glucocerebrosidase (GBA) and leucine-rich repeat kinase 2 against our subtypes. Then we calculated Genetic Risk Scores (GRS) for PD, multiple system atrophy, progressive supranuclear palsy, Lewy body dementia, and Alzheimer’s disease. These GRSs were regressed against the probability of belonging to a subtype in the two independent cohorts and we calculated q-values as an adjustment for multiple testing across four subtypes. We also carried out a Genome-Wide Association Study (GWAS) of belonging to a subtype.

**Results:**

A severe disease subtype had the highest rates of patients carrying GBA mutations while the mild disease subtype had the lowest rates (p=0.009). Using the GRS, we found a severe disease subtype had a reduced genetic risk of PD (p=0.004 and q=0.015). In our GWAS no individual variants met genome wide significance (<5×10e-8) although four variants require further follow-up, meeting a threshold of <1×10e-6.

**Conclusions:**

We have found that four previously defined PD subtypes have different genetic determinants which will help to inform future studies looking at underlying disease mechanisms and pathogenesis in these different subtypes of disease.

**WHAT IS ALREADY KNOWN ON THIS TOPIC**

- Data-driven approaches have been used to generate Parkinson’s disease subtypes in many studies but little is known about the genetics of these subtypes.

**WHAT THIS STUDY ADDS**

- We found in previously developed Parkinson’s subtypes that a severe disease subtype had the highest rates of glucocerebrosidase mutation carriers and the lowest genetic risk within a Parkinson’s Genetic Risk Score.

**HOW THIS STUDY MIGHT AFFECT RESEARCH, PRACTICE AND/OR POLICY**

- These results provide some biological validity to our data-driven subtyping approach and will assist in future studies looking at underlying disease mechanisms and pathogenesis.

## Introduction

Parkinson’s disease (PD) is a common and progressive neurodegenerative disorder encompassing a wide range of motor and non-motor features. There is considerable heterogeneity within these features in terms of presentation and progression which has led many to believe there are different clinically relevant subtypes of the disease. Data-driven approaches have been applied to many PD cohorts to try and delineate these subtypes, the first was in 1999^1^ and three systematic reviews have since been published.^2–4^ Other hypothesis driven approaches have also been studied in PD,^2 3^ the most commonly studied is the tremor-dominant (TD) versus postural instability gait difficulty (PIGD) motor subtype^5 6^ and another of interest is splitting into young-onset versus late-onset PD.^7 8^

We previously derived Parkinson’s clinical subtypes in over 2500 early patients with PD recruited from two large cohorts: Oxford Discovery and Tracking Parkinson’s.^9 10^ These subtypes were derived from the baseline motor and non-motor features using a data-driven approach, which were associated with subsequent motor progression and the medication response. We have recently shown, within the Oxford Discovery cohort, that one of our subtypes had a distinctive biomarker profile with reduced apolipoprotein A1 and increased C reactive protein levels, lending biological validity to our approach.^11^

Considering differences in genetics might help determine any difference in the aetiology of the subtypes while also providing a biological confirmation of data-driven clustering approaches. Here, we report on the genetics of our validated PD subtypes using data from the Oxford Discovery and Tracking Parkinson’s cohorts combined. To calculate the genetic risk of PD and related conditions including the atypical parkinsonian disorders and Alzheimer’s disease (AD), we identified a Genome Wide Association Study (GWAS) of disease status (an analysis of case/control status) for each of the diseases. We then looked at whether the genetic risk of PD and related disorders was associated with belonging to a particular disease subtype. We also considered two of the most important mutations in PD, glucocerebrosidase (GBA) and leucine-rich repeat kinase 2 (LRRK2), against our subtypes. Finally, we carry out a GWAS study to see whether any individual genetic variants are associated with belonging to a subtype.

A recent GWAS study has been published based on the TD and PIGD motor subtypes which found multiple PD risk alleles that might influence the motor subtype.^12^ We have recently published a GWAS study using data from the Oxford Discovery, Tracking Parkinson’s and PPMI cohorts to look at motor and cognitive progression which found that APOE ε4 influences progressive cognitive impairment.^13^ This study differs to our previous one as its focus is on data-driven PD subtypes.

## Methods

### Cohorts

We used data from two large prospective early PD cohorts. The Tracking Parkinson’s cohort includes UK-wide centres, recruited between February 2012 and May 2014. Full details of this cohort along with inclusion/exclusion criteria have been published previously.^14^ The Oxford Discovery cohort includes patients from 11 hospitals in the Thames Valley region recruited between September 2010 and January 2016.^15^ In both cohorts, patients were recruited within 3.5 years of diagnosis, and both studies were funded by Parkinson’s UK. Both studies had ethical approval and were undertaken with the understanding and written consent of each subject. Patients are followed up every 18 months collecting a wide range of data in motor, non-motor and cognitive domains. For brevity, we will refer to the Tracking Parkinson’s cohort as Tracking.

### Patient evaluation

Our data-derived PD subtypes were determined using variables from motor, non-motor and cognitive domains at baseline. Our clustering approach used a factor analysis followed by a k-means cluster analysis where we considered two to five clusters. Individuals were excluded from the cluster analysis if they had been rediagnosed with another condition during follow-up or if they had been given a probability of a diagnosis of PD of <90% at the latest visit as rated by a research neurologist or movement disorder specialist. This was an attempt to exclude those incorrectly diagnosed with PD.

Our first paper on this subject was based on only the Oxford Discovery cohort (with 769 patients) and we found five clusters gave us the optimal solution.^9^ In our second paper we used two cohorts where the Tracking cohort (n=1601) was chosen to be the development cohort (as it was larger) and the Oxford Discovery cohort (n=944) was the validation cohort.^10^ Here, we identified that four clusters were the optimal solution. Comparing the actual and predicted clusters (from a discriminant analysis model fitted to the Tracking clusters) in Oxford Discovery gave us a kappa statistic of 0.58 indicating moderate agreement, providing evidence our cluster approach was moderately stable across the two cohorts. These four clusters (derived using only baseline data) were shown to be associated with different subsequent motor progression over an average of 3 years follow-up and also with medication response using a levodopa challenge. We also found differences in age, gender, Hoehn and Yahr stage as well as TD/PIGD rates between the clusters which were all factors not included in the cluster analysis. The identified clusters were named (1) fast motor progression with symmetrical motor disease, poor olfaction, cognition and postural hypotension; (2) mild motor and non-motor disease with intermediate motor progression; (3) severe motor disease, poor psychological well-being and poor sleep with an intermediate motor progression and (4) slow motor progression with TD, unilateral disease. When we talk about mild/severe disease we are classifying the cross-sectional associations of data at baseline while fast/slow refers to progression rates after baseline so fast/severe and slow/mild can be thought of as different clusters. In this paper, we describe the genetics of the four subtypes (also referred to as clusters since they were developed using cluster analysis) from our development/validation paper.^10^ Within the Oxford Discovery cohort we report on the predicted clusters since any future research on individuals outside of these cohorts would rely on predictions.

### Genotyping

In the Tracking Parkinson’s cohort, individuals were genotyped using the Illumina HumanCore Exome array with custom content.^14^ Within the Oxford Discovery cohort individuals were genotyped on either the Illumina HumanCore Exome-12 V.1.1^16^ or the Illumina InfiniumCore Exome-24 V.1.1^17^ singl-nucleotide polymorphisms (SNP) arrays. The quality control and imputation of this data has been previously described^13^ and is also described in the online supplemental file 1.

10.1136/jnnp-2021-327376.supp1 Supplementary data

In a principal components (PCs) analysis, 20 genetic PCs were generated from a linkage-pruned SNP set (removing SNPs with an r^2^ >0.02 in a 1000 kb sliding window shifting 10 SNPs at a time). If an individual was >6 SDs from the mean of one of the first 5 PCs or a clear outlier in a scatter plot they were excluded and then the PCs recalculated and repeated until there were no outliers. The first five PCs were then retained to be included as covariates within the GWAS.

Our main focus was to look at genetic risk of PD but we also wanted to explore whether they might be shared genetic pathways between other neurodegenerative disorders (progressive supranuclear palsy (PSP), multiple system atrophy (MSA), Lewy body dementia (LBD) and AD) and each subtype while also exploring the potential for selection bias where atypical parkinsonian disorders might have been incorrectly diagnosed as PD. To calculate the genetic risk of each condition we identified an external GWAS of disease status (an analysis of case/control status) applied to separate PD, MSA, PSP, LBD and AD cohorts.^18–22^ Overlap in genetic pathways and risk has been described previously for LBD, Parkinsons and Alzheimer’s.^21^ The PD GWAS^19^ reports that applying a Genetic Risk Score (GRS) using the genome-wide significant hits explained a minimum of 16% of the genetic liability and led to an AUC of 0.651.

GBA mutations were split into those that are recognised as causing Gaucher’s disease (GD) (the most common being L444P and N370S) and those that are not (E326K and T369M) as previously reported from Tracking.^23^ For LRRK2, we identified carriers of the G2019S and R1441C mutations, as reported previously from Tracking.^24^ In Oxford Discovery, carriers of L444P and R1441C mutations were identified by PCR as previously reported^25^ and the other mutations were identified from the Neurochip^26^ which is a custom-designed array for the investigation of genetic variation in neurodegenerative diseases and can detect rare variants within the LRRK2 and GBA genes. The Neurochip data underwent similar Quality control to the array data described above and is also described in the online supplemental file 1. In Oxford Discovery, we have carried out Sanger sequencing to confirm the N370S and E326K mutation carriers. All those who underwent Sanger sequencing had the mutation confirmed, however, two of the N370S carriers have not yet had Sanger sequencing. The numbers with other monogenetic forms of PD such as PRKN, SNCA and PINK1 were too small to draw any conclusions, see discussion.

### Statistical analysis

We tabulated the clusters against LRRK2 and GBA status using a Fisher’s exact test (since the frequencies are very small in some cells due to the rarity of these mutations) to determine the strength of any association.

We calculated the probability of belonging to a cluster from the discriminant analysis model from our validated subtypes paper.^10^ This probability was converted to log odds to give a more suitable continuous score for linear regression (unbounded range and symmetrical).

In an attempt to assess the potential for selection bias we compared age (t-test), gender and cluster assignment (χ^2^ test) for those who did and did not have genetic data from the SNP arrays after quality control.

We calculated GRS for PD, PSP, MSA, LBD and AD by multiplying the genome wide significant SNPs (p<5×10e-8) by their beta coefficients taken from each external GWAS and then standardising the score. This GRS can be interpreted as an estimate of the contribution of genetics to developing one of these diseases.^27^ Since the MSA GWAS did not find any genome wide significant SNPs we used those reported at a threshold of <1×10e-6 to calculate the GRS^20^ and in Alzheimer’s we used two variants that were from previously reported genome-wide significant loci but did not reach significance in the current GWAS.^22^ The number of SNPs from each GRS are reported in online supplemental table 1, which are the number of SNPs reaching the thresholds specified above in each external GWAS that were also available in our genetic data. Then we used linear regression with log odds of belonging to a cluster as the outcome and each GRS as the exposure. This was carried out separately within each cohort and then the results were combined using a fixed effects meta-analysis. We used a false discovery rate method,^28^ often called the Benjamini-Hochberg method, to control for multiple comparisons across the four subtypes.^11^ Using this method in our GRS analyses we have derived q-values. If our significance threshold was 0.05 we would hope to find q values <0.05. These q-values do not have a simple probabilistic interpretation, it is only important whether they reach the chosen threshold. The authors are aware of problems using corrections to p values^29^ and focusing on statistical significance at an arbitrary 0.05 threshold.^30 31^ We have tried to not use language like significant and non-significant, instead p values should be viewed by the reader as a continuum where smaller p values represent greater evidence against the null hypothesis and confidence intervals should be examined for the strength of any association. In the results, we have pointed out the direction of some associations and using the derived p values and q-values the reader can decide for themselves the strength of evidence against the null hypothesis. We hope this approach will promote modern thinking that arbitrary p value thresholds are unhelpful.

We carried out a GWAS with linear regression using the logs odds of belonging to a cluster as the outcome. The first five genetic PCs were used as covariates for each regression. Only SNPs with a minor allele frequency (MAF) >0.05 were included. The data were combined using a fixed effects meta-analysis. We also computed the expected power for our sample size^32^ for a range of beta and MAFs. The number of SNPs within the GWAS are reported in the online supplemental file 1.

Palindromic SNPs (where the alleles are nucleotides that pair to each other making it difficult to determine the direction of effect) that had an MAF >0.45 were excluded when calculating the GRS and also from the GWAS.

## Results

### Demographics and potential for selection bias

After all the quality control procedures, we had genetic data on 1467 derived from 1601 (91.6%) individuals from the original Tracking cluster analysis. Average age (67.2 vs 68.0 with p=0.31) and gender rates (34.2% vs 34.3% female with p=0.97) were similar in those with and without genetic data (respectively). Looking within clusters rates of those included varied from 96.8% (cluster 4) to 88.6% (cluster 1) with a p=0.001. For those with genetic data there were 437, 423, 304 and 303 individuals in clusters 1–4, respectively.

In the Oxford Discovery cohort, we had genetic data on 807 individuals, out of 944 (85.5%) individuals from the cluster analysis. Within Oxford Discovery average age (67.4 vs 66.1 with p=0.15) and gender rates (34.3% vs 41.6% female with p=0.099) were similar in those with and without genetic data (respectively). Looking within clusters rates of those included varied from 87.5% (cluster 4) to 83.0% (cluster 3) with a p =value 0.53. For those with genetic data there were 261, 145, 185 and 216 individuals in clusters 1–4, respectively.

### Mutation carriers

Table 1 shows the associations between LRRK2 and GBA mutation carriers against the clusters in both cohorts. In the Tracking cohort the third cluster (severe motor disease and poor psychological well-being) had the largest proportion of LRRK2 carriers (1.9%), however, this is not replicated in Oxford Discovery where the third cluster has no carriers. The combined cohort p value of LRRK2 vs the clusters was p=0.35.

**Table 1:** Data-derived clusters compared with LRRK2 and GBA mutation status

	LRRK2			GBA		
	Non-carriers	Carriers		Non-carriers	E326K and T369M carriers	GD-causing variants
**Tracking Parkinson’s cohort**						
Cluster 1	469 (99.8%)	1 (0.2%)	Cluster 1	437 (91.8%)	29 (6.1%)	10 (2.1%)
Cluster 2	432 (99.1%)	4 (0.9%)	Cluster 2	413 (93.7%)	20 (4.5%)	8 (1.8%)
Cluster 3	314 (98.1%)	6 (1.9%)	Cluster 3	282 (87.0%)	27 (8.3%)	15 (4.6%)
Cluster 4	304 (99.7%)	1 (0.3%)	Cluster 4	280 (90.9%)	20 (6.5%)	8 (2.6%)
P=0.059			P=0.080			
			P value (GBA variants combined)=0.018			
**Oxford discovery cohort**						
Cluster 1	280 (99.3%)	2 (0.7%)	Cluster 1	231 (90.9%)	15 (5.9%)	8 (3.2%)
Cluster 2	150 (98.7%)	2 (1.3%)	Cluster 2	127 (93.4%)	8 (5.9%)	1 (0.7%)
Cluster 3	204 (100%)	0	Cluster 3	158 (88.8%)	14 (7.9%)	6 (3.4%)
Cluster 4	221 (99.1%)	2 (0.9%)	Cluster 4	185 (90.7%)	16 (7.8%)	3 (1.5%)
P=0.45			P=0.57			
			P value (GBA variants combined)=0.59			
Combined cohort						
Combined cohort p=0.35			Combined cohort p=0.036			
			Combined cohort p value (GBA variants combined)=0.009			

Note the numbers in this table are slightly different to the numbers in the other analyses since the mutation status did not come from the imputed array data.

GBA, glucocerebrosidase; GD, Gaucher’s disease; LRRK2, leucine-rich repeat kinase 2.

Within the Tracking cohort the third disease cluster (severe motor disease and poor psychological well-being) had the greatest proportion of GBA carriers (12.9% across both carrier groups) and the second disease cluster (mild motor and non-motor disease) had the lowest proportion of GBA carriers (6.3%). This trend was also seen in Oxford Discovery cohort (11.3% in cluster 3 vs 6.6% in cluster 2). In the combined cohorts a p value for a difference in GBA carrier rates across the clusters was p=0.036, and when combining the two GBA carrier groups the p value was smaller at p=0.009.

### Genetic risk of diseases

**Figure 1:**
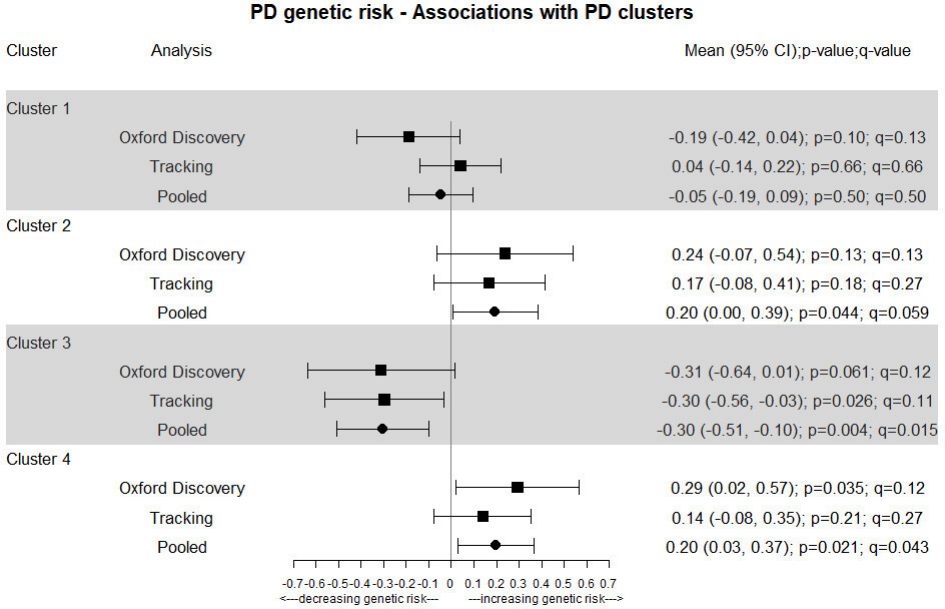
Genetic risk of Parkinson’s disease (PD) versus likelihood of belonging to a cluster.

Genetic PD risk (see figure 1) is positively associated with belonging to clusters 2 (mild motor and non-motor disease) (pooled p=0.044 and q=0.059) and 4 (slow motor progression), (pooled p=0.021 and q=0.043), while it is negatively associated with belonging to cluster 3 (severe motor disease and poor psychological well-being) (p=0.004 and q=0.015). For the pooled associations a one SD change in the PD GRS was associated with a 0.2 (95% CI 0.00 to 0.39) increase in the log odds of belonging to cluster 2; 0.2 (95% CI 0.03 to 0.37) increase for cluster 4 and a 0.3 (95% CI 0.10 to 0.51) decrease for cluster 3. We also explored a sensitivity analysis where we adjusted for the GBA mutation carrier groups and found very similar results (see online supplemental figure 1).

**Figure 2:**
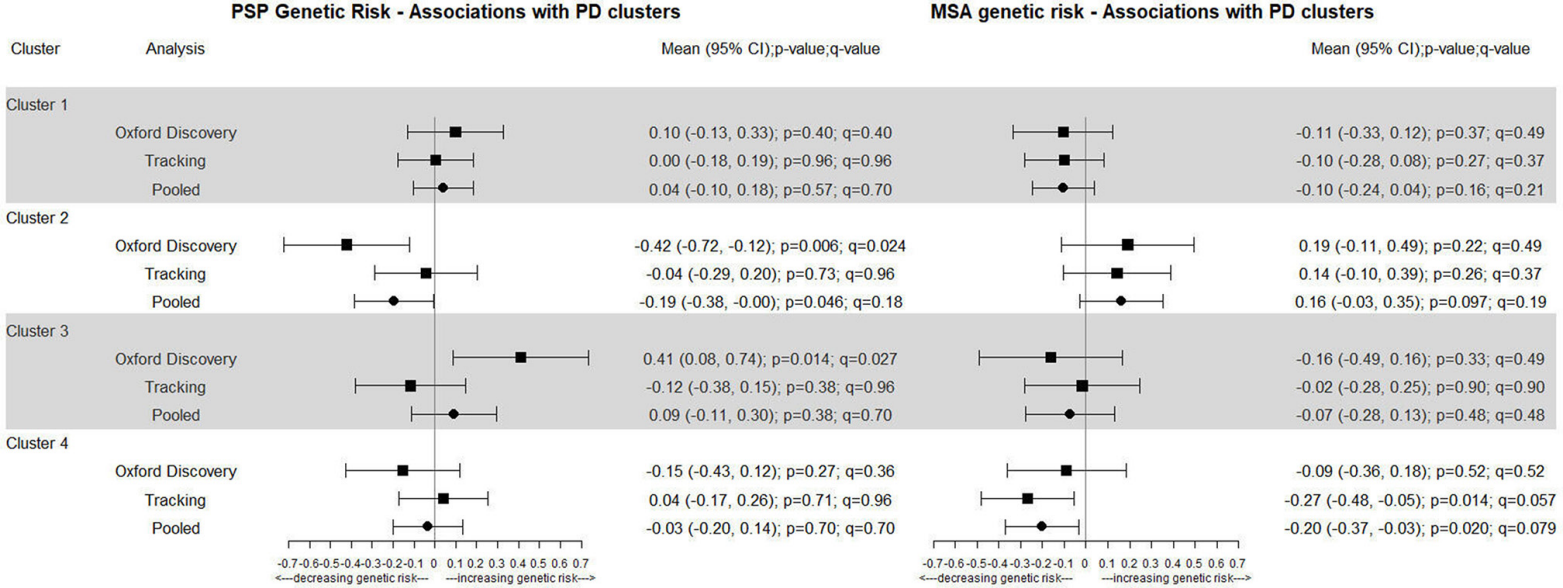
Genetic risk of atypical Parkinson’s: progressive supranuclear palsy (PSP) and multiple system atrophy (MSA).

We can see in figure 2 that within the Oxford Discovery cohort genetic PSP risk is negatively associated with cluster 2 (mild motor and non-motor disease) (p=0.006 and q=0.024) and positively associated with cluster 3 (severe motor disease and poor psychological well-being) (p=0.014 and q=0.027). However within the Tracking cohort the association between PSP with cluster 2 (mild motor and non-motor disease) is much smaller (-0.04 vs −0.42) and for cluster 3 (severe motor disease) it is within the opposite direction (-0.12 vs 0.41). When compared with the Oxford Discovery cohort the pooled p values and q values are much larger for both cluster 2 (p=0.046 and q=0.18) and cluster 3 (p=0.38 and q=0.70). Also within figure 2, we can see that genetic MSA risk is negatively associated with belonging to cluster 4 (slow motor progression) (pooled p=0.020 and q=0.079) where a 1 SD change in the GRS was associated with a 0.20 (95% CI 0.03 to 0.37) decrease in the log odds of belonging to cluster 4.

**Figure 3:**
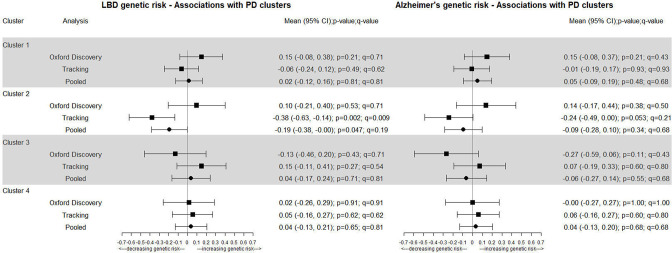
Genetic risk of dementia: Alzheimer’s disease and Lewy body dementia (LBD). PD, Parkinson’s disease.

In figure 3, we can see that the associations of the clusters with genetic risk of LBD and AD look very similar (especially for clusters 1, 2 and 4). Cluster 2 (mild motor and non-motor disease) is inversely associated with both LBD and AD in Tracking but not within Oxford Discovery. Within the AD GWAS the APOE genetic variant has an effect size much higher than all the others (OR of 3.32 compared with an average of 1.27 when the direction of effect is coded as positive) so we also explored what would happen when that variant is removed (see online supplemental figure 2). When removing this variant cluster 1 (fast motor progression) is positively associated with AD (pooled p=0.063 and q=0.25) where a one SD change in the GRS was associated with a 0.13 (−0.01 to 0.28) increase in the log odds of belonging to cluster 1.

### Genome Wide Association Study

There was little evidence of population stratification since within the four GWAS analyses from Tracking, the genomic inflation factor lambda varied from 1.001 to 1.008, while within Oxford Discovery they were all 1.0.

We highlight the power we have to detect a genome wide significant variant given our sample size in online supplemental table 2. Generally our power is small to detect rare variants with high effect sizes or common variants with small effect sizes. Since we found no genome wide significant variants in table 2 we highlight (non-independent) variants that reached a threshold of <1×10e-6, similar to the MSA GWAS study.^20^ At this threshold we identified 3 SNPs that were associated with cluster 1. The QQ-plot for this cluster (see online supplemental figure 3) shows a hump at the upper end which implies an excess of genetic variants associated with phenotypic cluster 1 at lower p value levels (0.0001–0.000001). We had one SNP at the reduced threshold for cluster 3 and none for clusters 2 and 4. None of the other QQ-plots (see online supplemental figures 4–6) show evidence of there being an excess of variants associated with any phenotypic cluster. The cohort specific results from table 2 can be found in online supplemental table 3). In the online supplemental file 1 the biological relevance of the identified SNPs are reported along with some network analyses (none of which met a threshold of Bonferroni adjusted-value of 0.05 shown in online supplemental figures 7–10).

**Table 2:** SNPs meeting a threshold of 1×10e-6 from the genome wide association study meta-analysis for each data-driven cluster

Chr	Position (GRCh37)	Marker	A1	A2	Nearest gene	Beta	SE	P value
**Cluster 1**								
1	237 734 615	rs151043031	CT	C	RYR2	0.59	0.12	9.986e-07
6	160 698 177	rs316037	G	A	SLC22A2	0.60	0.12	9.867e-07
6	160 699 605	rs5881357	AT	A	SLC22A2	0.60	0.12	6.337e-07
**Cluster 2—no SNPs met threshold**								
**Cluster 3**								
1	214 449 747	rs116258323	T	C	SMYD2	1.62	0.33	6.715e-07
**Cluster 4—no SNPs met threshold**								

A1, effect allele; A2, other allele; Chr, chromosome; SE, SE error; SNPs, single-nucleotide polymorphisms.

## Discussion

The associations between GBA and the phenotypic clusters, with a severe disease cluster having the greatest proportion of carriers and a mild disease cluster having the smallest proportion, are what would be expected given the observational evidence that GBA mutations are associated with higher Hoehn and Yahr stage and worse cognition.^33–36^ GD-causing and GBA risk variants such as E365K (E326K) have also been associated with more rapid motor and cognitive impairment in PD in other studies.^37^ This has been hypothesised to relate to lysosomal dysfunction and the more rapid accumulation of pathogenic alpha-synuclein species in patients with carrying GBA variants.^38^ However, there are also reports that GBA mutations are associated with earlier disease onset while cluster 3 has the most GBA mutations and a higher than average age at diagnosis and cluster 2 has the least GBA mutations and the lowest average age at diagnosis.^10^ This highlights that there is still heterogeneity of disease onset within the clusters and that GBA mutation carriers are only a small proportion (~12%) of even the cluster with the highest carrier rate. We hypothesise that other similar genetic variants are associated with the severe disease cluster that may relate to impaired proteostasis and/or lysosomal dysfunction.

There is also heterogeneity of clinical phenotype within LRRK2 carriers which would make it difficult to correlate them with clusters. One study showed that mutations of the LRRK2 gene are associated with less cognitive impairment compared with iPD^39^ while others have failed to confirm this.^40 41^ A study of LRRK2 found a slower decline in UPDRS scores^42^ and another found no discernible effect on rate of motor disease progression.^43^

There are several possible explanations for the negative association between genetic risk of PD and the third, severe disease cluster. The first is that the individuals in this cluster have a more environmental and less genetically driven disease aetiology. The second is that this cluster is enriched with non-PD cases although the MSA and PSP genetic risk pooled associations do not support this, and it would also require that the PD GWAS studies had no enrichment of other similar conditions. The third is one of selection bias, in that these severe disease cases are less likely to participate in the PD cohorts that supply cases to the PD GWAS study we used, as compared with Oxford Discovery and Tracking cohorts which offered local clinical review for the majority of research participants. This PD GWAS study used data from 17 different datasets.^19^ Note that the GRS came from the imputed genetic data which excludes rarer genetic variants such as those within the GBA gene. The severe disease cluster has low genetic risk of PD looking at common variants yet the rare GBA variants have the highest frequency within this cluster.

We have data on other monogenetic forms of Parkinson’s (SNCA, PRKN and PINK1) and have published this data from the Tracking cohort.^24^ However, the numbers are too small to draw any conclusions against our clusters. Only one individual from the Tracking clusters have a biallelic PINK1 mutation, none had a biallelic PRKN mutation and only one a SNCA mutation. In the Oxford Discovery cohort, we have data within these genes from the Neurochip but again the numbers are too small to draw any conclusions, no one from the cluster analysis had a SNCA or a biallelic PRKN mutation and only one individual had a biallelic PINK1 mutation.

The negative association between genetic risk of PSP and cluster 2 and the positive association with cluster 3 in the Oxford Discovery cohort is what we would expect to see if there was enrichment of PSP cases. That is, PSP cases are more likely to belong to a severe motor disease cluster than a mild motor and non-motor disease cluster. However, this is not backed up by the associations within the larger Tracking cohort. This could represent a chance finding in Oxford Discovery. Alternatively, it could reflect the procedure we used to exclude patients from the analysis, that is dropping those with probability of diagnosis of PD of <90% at the latest clinic visit. In Tracking 367/1975 (18.6%) were dropped, while in Oxford Discovery only 76/1022 (7.4%) were dropped using this criterion (see online supplemental figure 1 in the original paper^10^). Since a greater proportion were dropped in Tracking it is more likely that we have excluded PSP cases from this cohort. The reported PD disease probability would, in all likelihood, be reduced if the clinician documented features consistent with atypical parkinsonism during the clinical review, including the presence of symmetrical motor disease, early onset falls, suboptimal levodopa response, a supranuclear gaze palsy or early autonomic failure.

In previous research, we found cluster 3 was associated with a higher proinflammatory baseline profile (raised CRP, reduced apolipoprotein A1). This is interesting, as it suggests that in PD subtype 3—who have greater rates of cognitive dysfunction—early immune modulation might improve clinical outcomes, for example, by reducing future dementia risk if commenced early enough in the disease process. The lower overall genetic risk of PD and a higher pro-inflammatory profile in this cluster, are consistent with a hypothesis that the aetiology of this cluster is more driven by environmental rather than genetic risk factors.

Although none of our individual variants met the GWAS p value significance threshold the ones that we highlight might be interesting for future follow-up and research. It could be that the variants, or closest genes to these variants, are a reason that a person develops a particular subtype of Parkinson’s.

In previous research, we used multinomial logistic regression to look at how blood biomarkers are associated with an individual belonging to one of the clusters.^11^ For this genetic analysis, we decided to simplify the analyses by carrying out four separate analyses using the probability of belonging to the cluster as the outcome. This made the GWAS easier to run and interpret with fewer variables to estimate.

The strengths of this study are we have used two large early in the disease course and well-phenotyped PD cohorts. Our subtypes were created using large amounts of phenotypic data incorporating 21 variables across 12 important domains and these subtypes were developed and validated in over 2500 subjects. These subtypes were shown to be associated with both motor progression and medication response in a levodopa challenge. The limitations of this study are that in terms of searching for individual genetic variants it is still too small to find any that reach genome wide significance, assuming that such variants exist. Also there is the possibility of selection bias as rates of those with genetic data varied by cluster within the Tracking cohort. The frequency of PD subtypes in our cohorts may be different to that in the general PD population if belonging to a subtype was related to agreeing to take part in our cohorts or our cohorts failed to identify specific individuals during recruitment. However, to bias our estimates of genetics versus the clusters, it would require that selection into our cohorts was also related to an individual’s genetics. Diagnosis of PD will not be perfect and some patients will turn out to have other parkinsonian disorders, although we have attempted to mitigate this by excluding individuals with a diagnostic probability of PD <90% at the latest visit.

There are other subtypes that have been defined by a data-driven cluster analysis on motor and non-motor symptomatic data. Currently, it is difficult to determine whether the cluster definition we have used is more robust or superior to other definitions. However, in a recent systematic review our paper was rated (among 25 other data-driven studies) along with two others as having the highest methodological quality and clinical applicability.^2^ What sets our cluster definition apart is our use of an external validation.

Future work is now ongoing to understand the underlying disease pathophysiology driving these different clinical clusters in early PD, and their subsequent progression. This will use a mechanistic approach comparing lysosomal, mitochondrial, inflammatory function, α-synuclein (α-syn) seeding amplification^44^ and α-omics profiles across the four PD clinical clusters.

The differences in genetics between these clusters lends biological validity to our data-driven clustering approach while also providing evidence that the different subtypes can inform on underlying disease mechanisms and pathogenesis, as well as informing individual disease trajectories in PD.

## Data Availability

Data are available on reasonable request. Data from the Oxford Discovery cohort is available on request from https://www.dpag.ox.ac.uk/opdc/research/external-collaborations. Data from the Tracking Parkinsons cohort is available on request from https://www.trackingparkinsons.org.uk/about-1/data/.
