# Supplementary material for: Genetics of validated Parkinson’s disease subtypes in the Oxford Discovery and Tracking Parkinson’s cohorts
Source: J Neurol Neurosurg Psychiatry. 2022 Jun 21;93(9):952–9. doi: 10.1136/jnnp-2021-327376 (PMC9380504; doi:10.1136/jnnp-2021-327376)
Supplement: Supplementary data [file jnnp-2021-327376supp001.pdf]

Web-appendix

Genetics of validated Parkinson's Disease subtypes in the Oxford Discovery and Tracking Parkinson's cohorts

Lawton M, *et al. J Neurol Neurosurg Psychiatry* 2022; 93:952–959. doi: 10.1136/jnnp-2021-327376

Illumina Array quality control and Imputation

The same standard quality control procedures on the genotype data were carried out separately within the two cohorts. Individuals were excluded for the following reasons: related individuals as identified by an Identity-By-Descent PIHAT > 0.1; individuals with low overall genotyping rates (<98%); heterozygosity outliers, where data was >2 standard deviations away from the mean; and individuals whose reported sex was not the same as the genetically determined sex. A principle components analysis, after merging with European (CEU) samples from the HapMap reference panel, was carried out and individuals who were >6 standard deviations away from the mean of any of the first 10 principle components (PC) were also excluded. Genetic variants were excluded if they had a low genotyping rate (<99%), minor allele frequency <1% or a Hardy-Weinberg equilibrium p-value < $5x\ 10^{-6}$.

After these quality control steps the genotypes were imputed, separately by cohort, to the 1,000 Genomes Project reference panel (phase 3 release 5)[1] using the Michigan Imputation Server (https://imputationserver.sph.umich.edu). Finally genetic variants were excluded if the imputation quality scores (R2) were < 0.8 and then imputation dosages were converted into hard call genotypes. After the imputation and quality control there were 9,153,714 SNPs in the Oxford Discovery cohort and 8,986,152 SNPs in the Tracking cohort. When we carried out the pruning to generate PC's in the two cohorts there were 25,647 SNPs in the Oxford Discovery cohort and 23,876 SNPs in the Tracking cohort.

Lawton M, *et al. J Neurol Neurosurg Psychiatry* 2022; 93:952–959. doi: 10.1136/jnnp-2021-327376

Neurochip quality control

Individuals were excluded when: low overall genotyping rates (<97%); heterozygosity outliers, data was >3 standard deviations away from the mean; and individuals whose reported sex was not the same as the genetically determined sex.

Number of SNPs in GWAS study

After excluding variants with a minor allele frequency (MAF) < 0.05 there were 6,625,590 variants remaining in the Oxford Discovery cohort of which another 76,381 were excluded due to being palindromic with a MAF > 0.45. So the final Oxford Discovery cohort GWAS included 6,549,209 variants.

After excluding variants with a minor allele frequency (MAF) < 0.05 there were 6,672,212 variants remaining in the Tracking cohort. Of these 34 were duplicates (with identical Chromosome number and base position) and 76,521 were palindromic. The final Tracking cohort GWAS included 6,595,657 variants.

In the final meta-analysis there were 6,413,412 variants.

Potential biological relevance of GWAS variants

The two SNPs from chromosome 6 (rs316037 and rs5881357) that were associated with belonging to cluster 1 are both significant expression quantitative trait loci (eQTL) for the gene *LPAL2* and also are significant splicing quantitative trait loci (sQTL) for the gene *SLC22A3* according to GTEx Portal on 22/09/2021 (dbGaP Accession phs000424.v8.p2). *SLC22A3* has been shown in humans to be related to choline metabolism in cancer (KEGG T01001: 6581 (genome.jp)) and *LPAL2* is related to lipoprotein (a) in humans (LPAL2 lipoprotein(a) like 2, pseudogene [Homo sapiens (human)] - Gene - NCBI (nih.gov)). The SNP rs5881357 is in the same genomic locus as SNPs that have been reported to be related to BMI [2], various blood biomarkers such as apolipoprotein B levels and lipoprotein (a) levels [3] and mean spheric corpuscular volume [4]. There is also some evidence that the *SLC22A3-LPAL2-LPA* gene cluster contributes to risk and severity of coronary artery disease [5, 6].

The SNP from chromosome 1 (rs116258323) that was associated with belonging to cluster 3 is a significant cis-eQTL for the gene *SMYD2* [7]. This gene has been shown in humans to be involved in lysine degradation and metabolic pathways (KEGG T01001: 56950 (genome.jp)) and is a promising candidate for the treatment of cardiovascular disease and cancer [8].

Looking at the 1000 genomes project (European population) the minor allele frequencies (MAF) for these SNPS were 0.207 (rs316037), 0.217 (rs5881357) and 0.068 (rs116258323). The MAF was not reported for rs151043031. These values are very similar to the MAFs in our data, see supplementary table 2, which might suggest that although these SNPs are not associated with Parkinson's as a whole they might be associated with specific subtypes.

Network analyses

Functional mapping and annotation of GWAS results were carried out using the freely available internet platform FUMA (https://fuma.ctglab.nl/) using standard settings [9]. Within FUMA, summary statistics from our four GWAS results were analysed using Multi-marker Analysis of GenoMic Annotation (MAGMA) gene property tests to compare enrichment of the average gene expression within different tissues. The resulting tissue expression enrichment for each cluster are within supplementary figures 7 to 10, the upper graph shows a tissue expression analysis on 30 general tissue types and the lower graph 53 specific tissue types. After Bonferroni correction there was no evidence of association between our GWAS results for any cluster and average gene expression per tissue.

REFERENCES

[1] C. Genomes Project, A. Auton, L.D. Brooks, et al., A global reference for human genetic variation, Nature 526(7571) (2015) 68-74.
[2] A.E. Locke, B. Kahali, S.I. Berndt, et al., Genetic studies of body mass index yield new insights for obesity biology, Nature 518(7538) (2015) 197-206.
[3] N. Sinnott-Armstrong, Y. Tanigawa, D. Amar, et al., Genetics of 35 blood and urine biomarkers in the UK Biobank, Nat. Genet. 53(2) (2021) 185-194.
[4] D. Vuckovic, E.L. Bao, P. Akbari, et al., The Polygenic and Monogenic Basis of Blood Traits and Diseases, Cell 182(5) (2020) 1214-1231 e11.
[5] L. Wang, J. Chen, Y. Zeng, et al., Functional Variant in the SLC22A3-LPAL2-LPA Gene Cluster Contributes to the Severity of Coronary Artery Disease, Arterioscler. Thromb. Vasc. Biol. 36(9) (2016) 1989-96.
[6] D.A. Tregouet, I.R. Konig, J. Erdmann, et al., Genome-wide haplotype association study identifies the SLC22A3-LPAL2-LPA gene cluster as a risk locus for coronary artery disease, Nat. Genet. 41(3) (2009) 283-5.
[7] U. Vosa, A. Claringbould, H.J. Westra, et al., Large-scale cis- and trans-eQTL analyses identify thousands of genetic loci and polygenic scores that regulate blood gene expression, Nat. Genet. 53(9) (2021) 1300-1310.
[8] X. Yi, X.J. Jiang, Z.M. Fang, Histone methyltransferase SMYD2: ubiquitous regulator of disease, Clin. Epigenetics 11(1) (2019) 112.
[9] K. Watanabe, E. Taskesen, A. van Bochoven, et al., Functional mapping and annotation of genetic associations with FUMA, Nat Commun 8(1) (2017) 1826.

Supplementary table 1. Number of SNPs included in each genetic risk score after excluding SNPs in quality control/imputation and those that were palindromic with a minor allele frequency > 0.45.

Analysis (total SNPs reported)	Tracking cohort	Discovery cohort
PD (90)	86	85
PSP (5)	5	5
MSA (23)	21	21
LBD (5)	5	5
AD (23)	22	21

PD = Parkinson’s Disease; PSP = progressive supranuclear palsy; MSA = multiple system atrophy; AD = Alzheimer’s disease; LBD = Lewy body dementia (LBD)

Supplementary table 2. Power calculations for a range of beta's and minor allele frequencies (MAF) for a sample size of 2274 (combined Discovery and Tracking) and p-value < 5x10e-8 . The beta's are in s.d. units for the outcome and the s.d of the outcome was 3.4, 4.6, 4.9 and 4.1 for clusters 1 to 4 respectively. For context the highest beta in s.d. units for cluster 1 was ~0.17 with a MAF of ~0.23 and the highest beta in s.d units for cluster 3 was ~0.33 with a MAF of ~0.05.

Beta\MAF	0.05	0.1	0.15	0.2	0.25	0.3	0.35	0.4	0.45	0.5
**0.1**	0.0%	0.0%	0.1%	0.3%	0.6%	0.9%	1.3%	1.6%	1.8%	1.9%
**0.15**	0.1%	0.8%	3.4%	8.2%	14.6%	21.4%	27.4%	32.0%	34.8%	35.8%
**0.2**	0.6%	8.2%	27.1%	49.1%	66.8%	78.3%	85.1%	88.9%	90.8%	91.3%
**0.25**	3.9%	35.8%	73.2%	91.3%	97.4%	99.1%	99.7%	99.8%	99.9%	99.9%
**0.3**	15.3%	74.8%	96.8%	99.7%	100.0%	100.0%	100.0%	100.0%	100.0%	100.0%
**0.35**	39.1%	95.6%	99.9%	100.0%	100.0%	100.0%	100.0%	100.0%	100.0%	100.0%
**0.4**	68.2%	99.7%	100.0%	100.0%	100.0%	100.0%	100.0%	100.0%	100.0%	100.0%

Supplementary Table 3. SNPs meeting a threshold of 1x10e-6 from the genome wide association study meta-analysis – cohort specific results.

**CLUSTER 1 - TRACKING COHORT**									
**Chr**	**Position (GRCh37)**	**Marker**	**A1**	**A2**	**N**	**A1 frequency**	**Beta**	**SE**	**p -value**
1	237734615	rs151043031	CT	C	1467	0.231	0.70	0.15	6.30e-06
6	160698177	rs316037	G	A	1467	0.217	0.54	0.16	0.000760
6	160699605	rs5881357	AT	A	1467	0.221	0.52	0.16	0.000875
**CLUSTER 1 - DISCOVERY COHORT**									
**Chr**	**Position (GRCh37)**	**Marker**	**A1**	**A2**	**N**	**A1 frequency**	**Beta**	**SE**	**p -value**
1	237734615	rs151043031	CT	C	807	0.230	0.42	0.19	0.0308
6	160698177	rs316037	G	A	807	0.214	0.70	0.19	0.000333
6	160699605	rs5881357	AT	A	807	0.224	0.72	0.19	0.000168
**CLUSTER 3 - TRACKING COHORT**									
**Chr**	**Position (GRCh37)**	**Marker**	**A1**	**A2**	**N**	**A1 frequency**	**Beta**	**SE**	**p -value**
1	214449747	rs116258323	T	C	1467	0.050	1.40	0.43	.00120
**CLUSTER 3 - DISCOVERY COHORT**									
**Chr**	**Position (GRCh37)**	**Marker**	**A1**	**A2**	**N**	**A1 frequency**	**Beta**	**SE**	**p -value**
1	214449747	rs116258323	T	C	807	0.058	1.92	0.50	.000129

Lawton M, *et al. J Neurol Neurosurg Psychiatry* 2022; 93:952–959. doi: 10.1136/jnnp-2021-327376

Supplementary figure 1. Genetic risk of Parkinson's when adjusting for GBA mutation carriers – sensitivity analysis

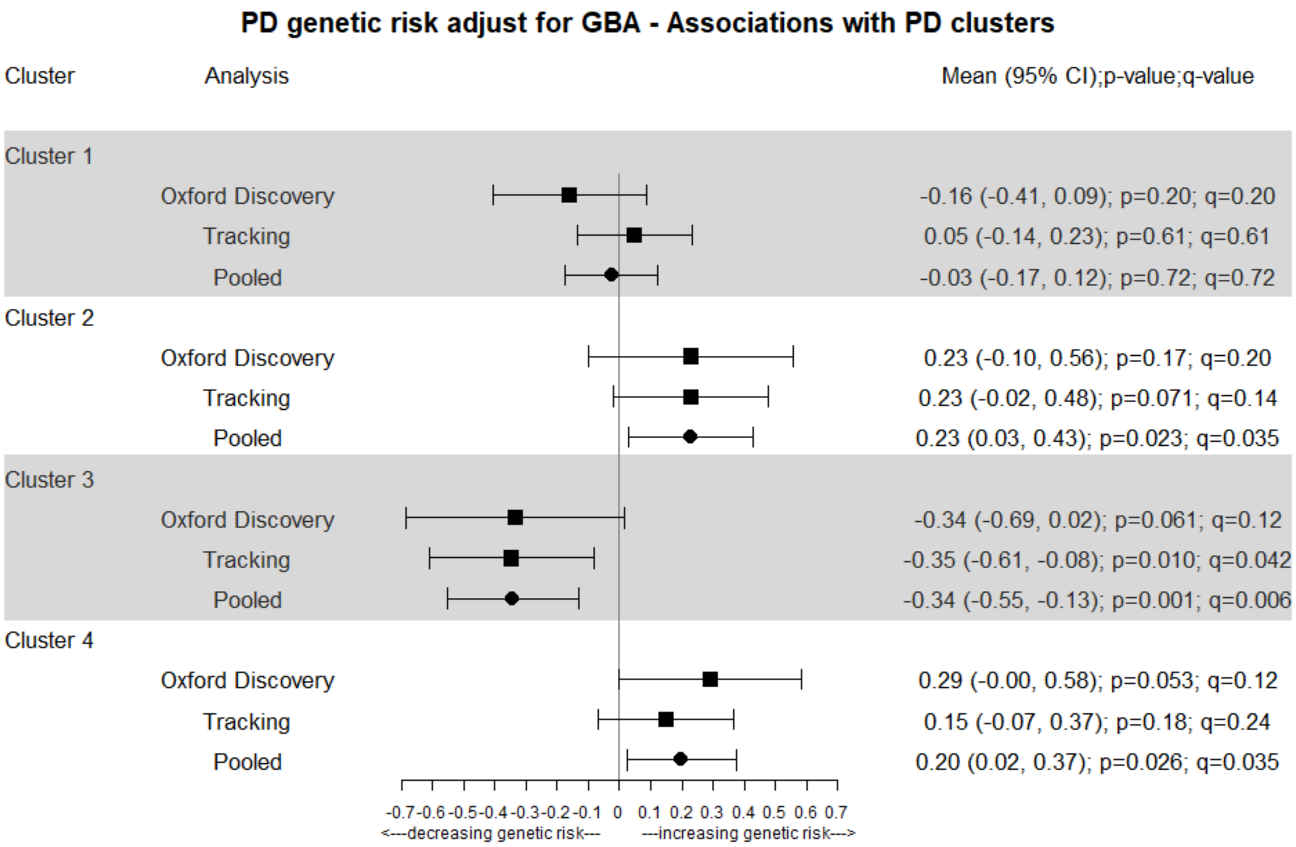

Supplementary figure 2. Genetic risk of Alzheimer's when removing the APOE genetic variant – sensitivity analysis

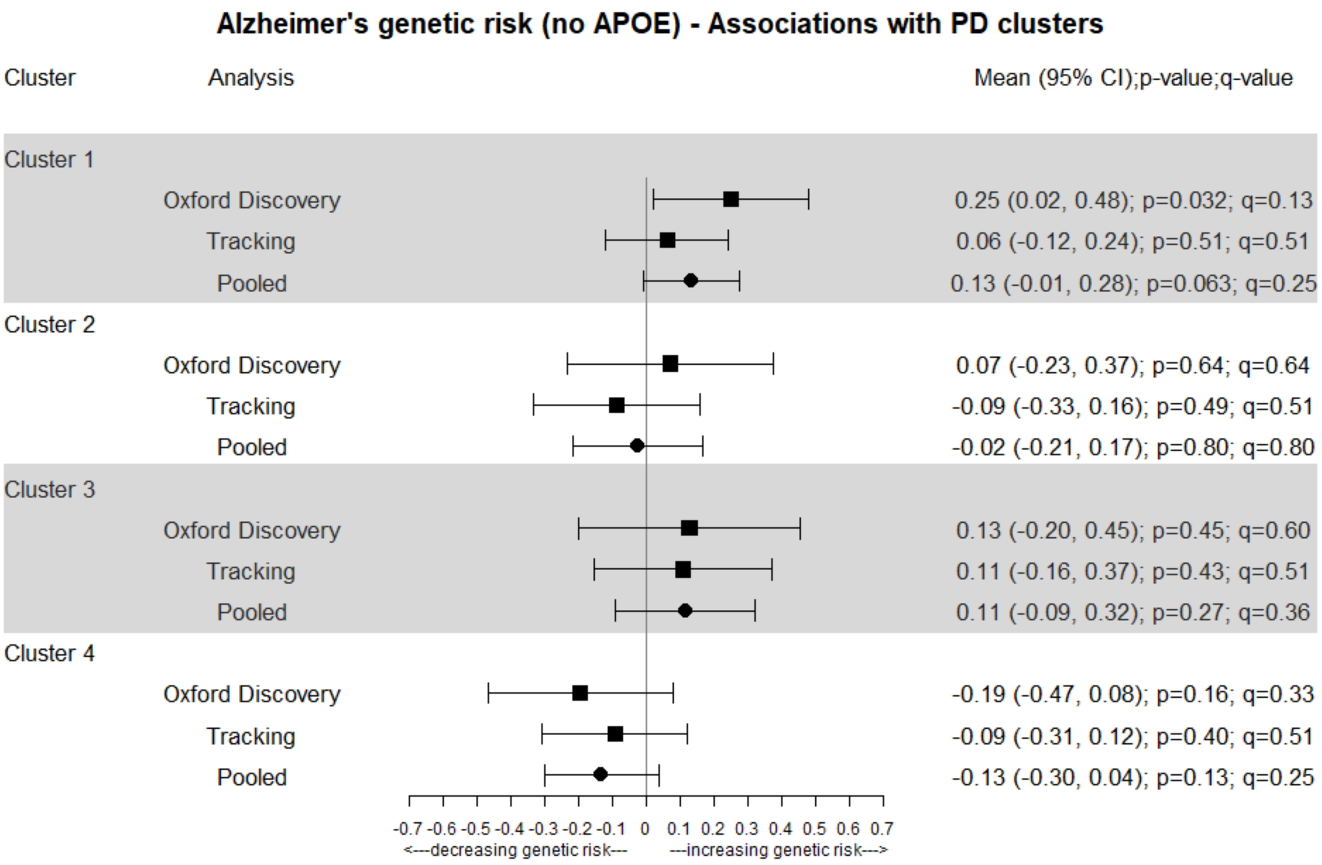

Supplementary figure 3. Manhattan Plot and QQ-plot for cluster 1 genome wide association study

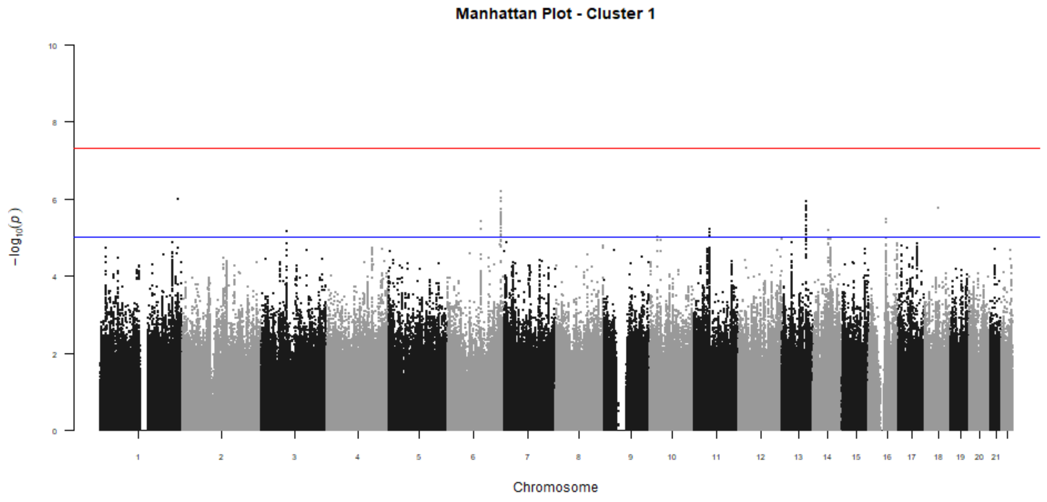

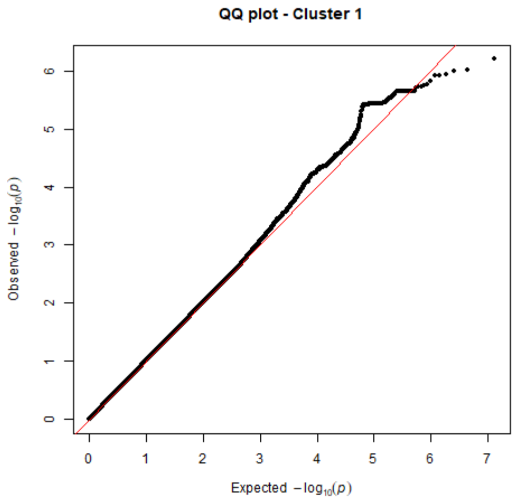

Lawton M, *et al. J Neurol Neurosurg Psychiatry* 2022; 93:952–959. doi: 10.1136/jnnp-2021-327376

Supplementary figure 4. Manhattan Plot and QQ-plot for cluster 2 genome wide association study

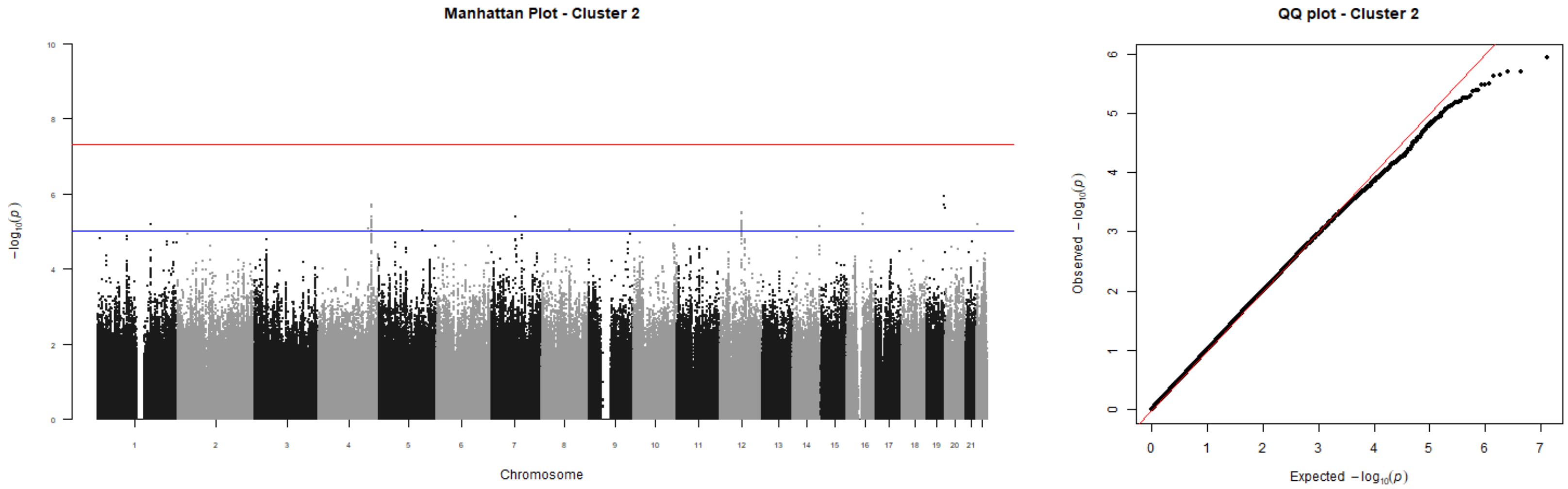

Supplementary figure 5. Manhattan Plot and QQ-plot for cluster 3 genome wide association study

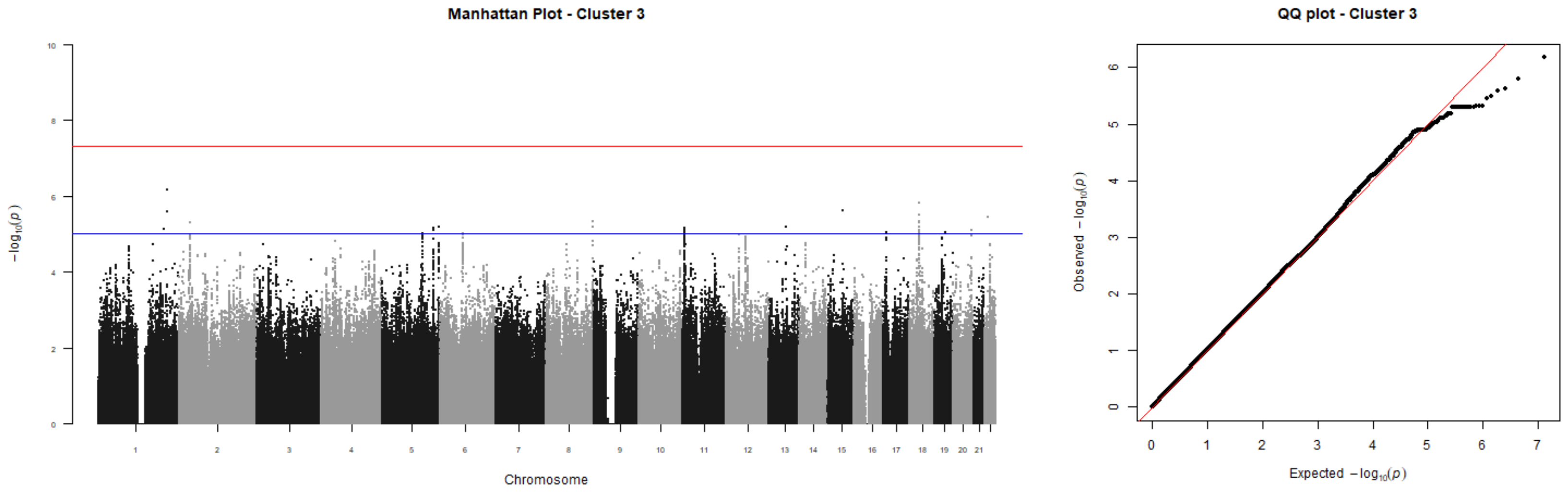

Supplementary figure 6. Manhattan Plot and QQ-plot for cluster 4 genome wide association study

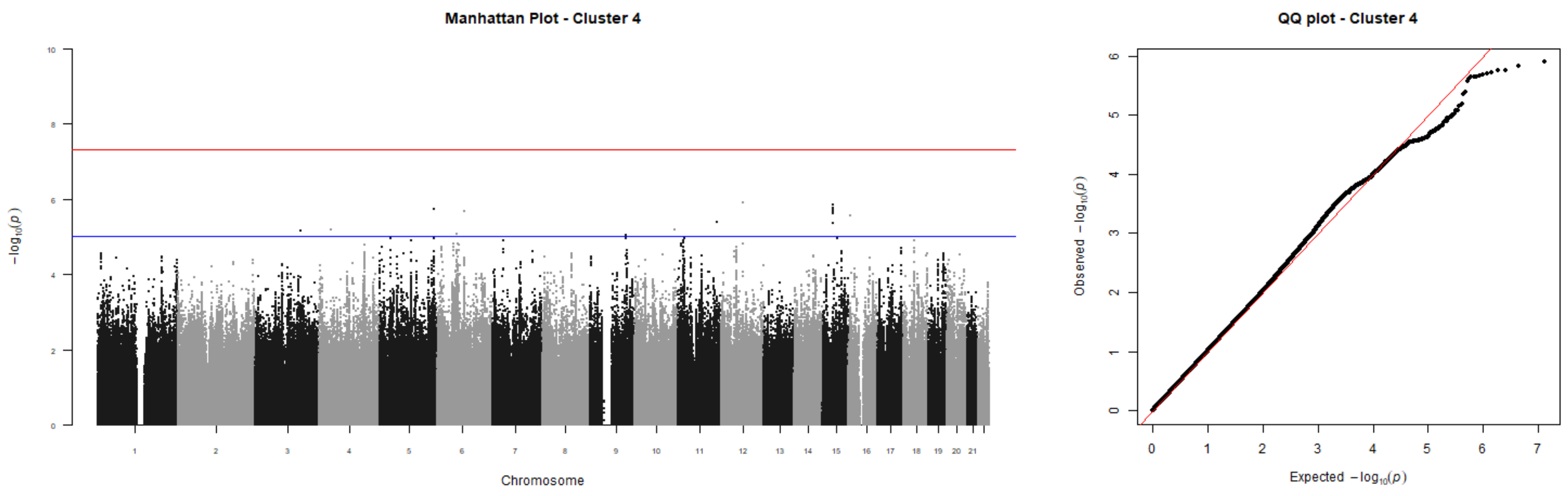

Supplementary figure 7. Results of FUMA analysis for MAGMA tissue expression analysis for cluster 1. Red bars would indicate a significance threshold of <0.05 for a Bonferroni adjusted p-value.

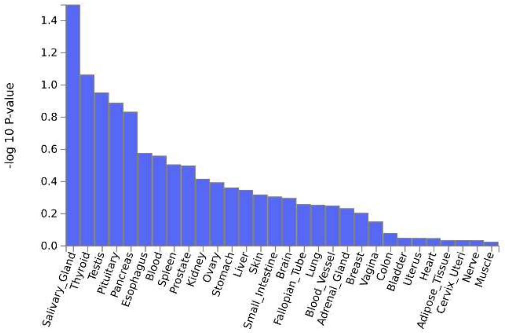

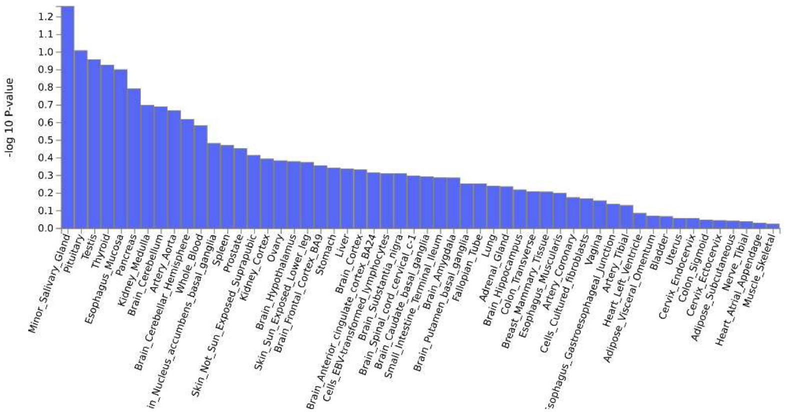

16

Genetics PD subtypes – web appendix

Supplementary figure 8. Results of FUMA analysis for MAGMA tissue expression analysis for cluster 2. Red bars would indicate a significance threshold of <0.05 for a Bonferroni adjusted p-value.

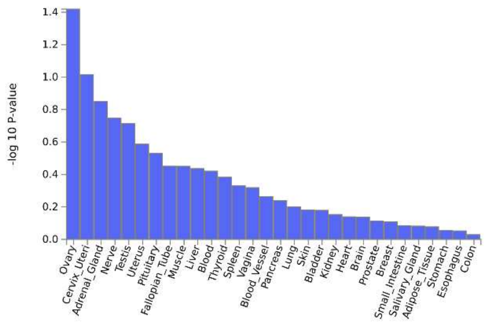

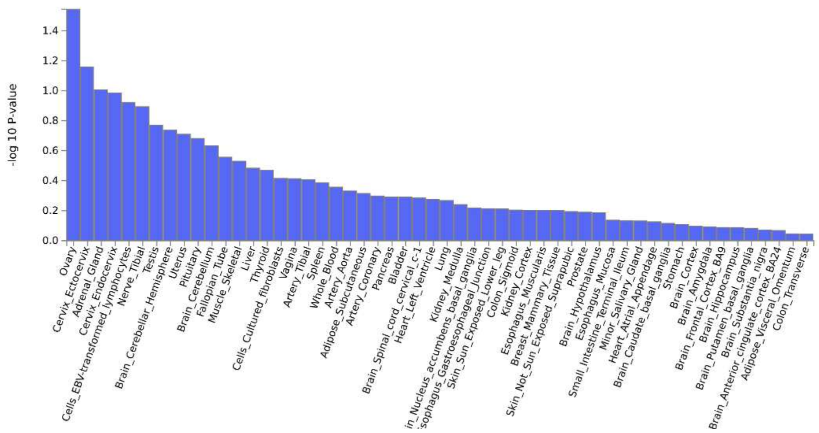

Supplementary figure 9. Results of FUMA analysis for MAGMA tissue expression analysis for cluster 3. Red bars would indicate a significance threshold of <0.05 for a Bonferroni adjusted p-value.

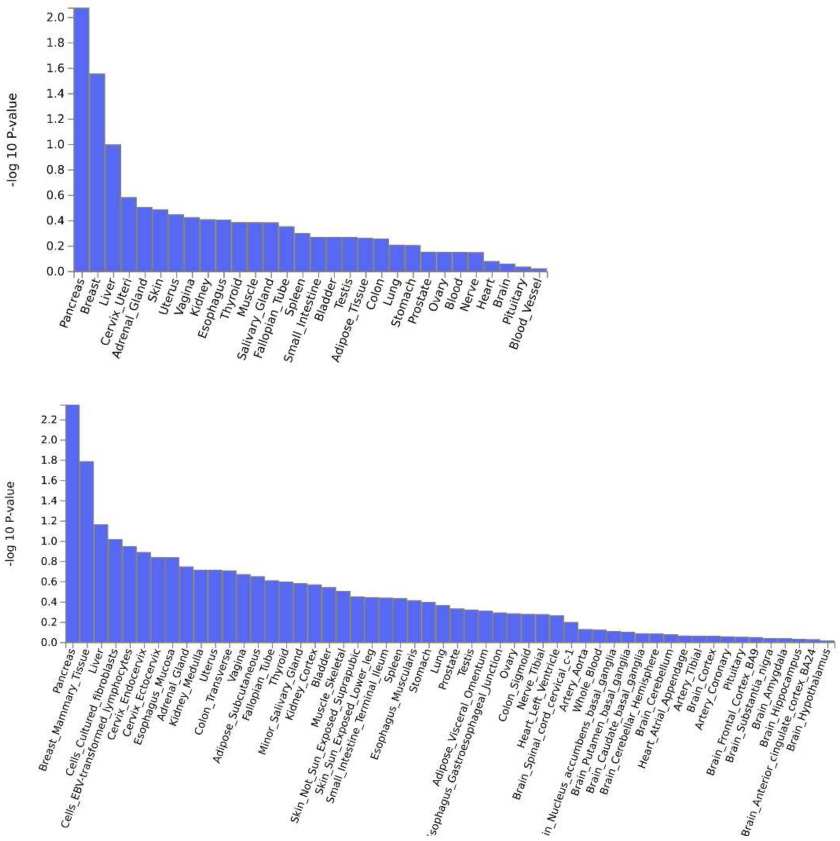

18

Genetics PD subtypes – web appendix

Supplementary figure 10. Results of FUMA analysis for MAGMA tissue expression analysis for cluster 4. Red bars would indicate a significance threshold of <0.05 for a Bonferroni adjusted p-value.

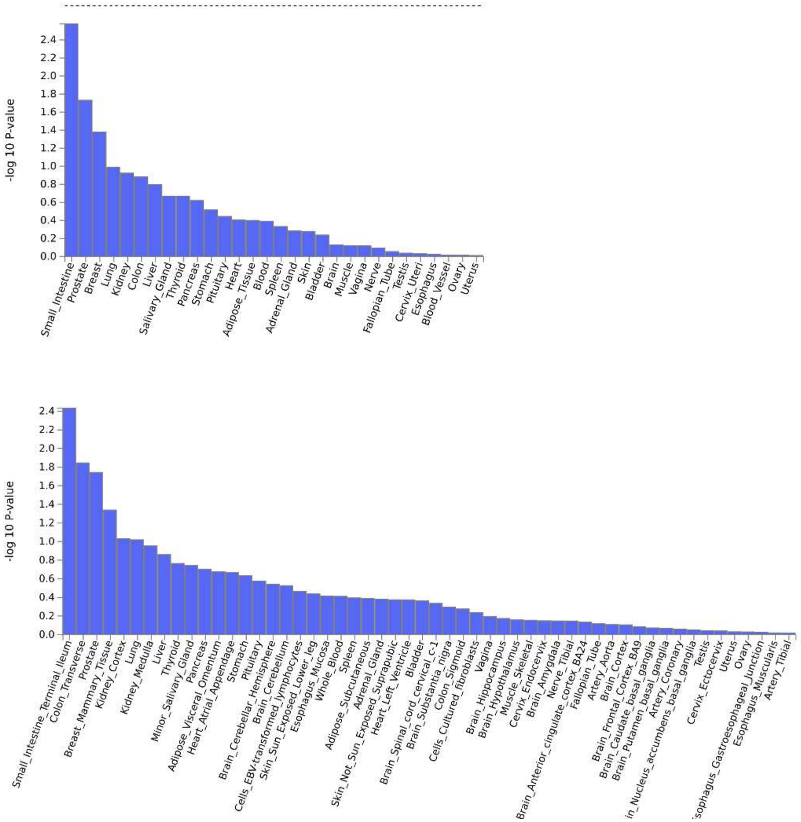

19

Genetics PD subtypes – web appendix
